# A journey to accreditation: is ISO 15189 laboratory accreditation possible in Ethiopia?

**DOI:** 10.11604/pamj.2016.24.329.8522

**Published:** 2016-08-30

**Authors:** Abay Sisay Misganaw

**Affiliations:** 1 Managment Sciences for Health, Heal TB, Addis Ababa, Ethiopia

**Keywords:** Laboratory, accerditation, journey, ISO 15189, Ethiopia

## Opinion

Laboratory accreditation system is found to be essential to have national and international acceptance of various laboratory test results [1]. In Ethiopia after the launching of WHO-AFRO Stepwise Laboratory Accreditation Process in 2009, laboratories were selected for accreditation purpose by the national and the only lab score 4 star based on WHO AFRO assessment checklist is Addis Ababa Health Research& Laboratory (AAHRL)(Nov. 2011) from a baseline of 0-star [2]. This makes the Addis Ababa health Research & Laboratory management & staffs to take the initiative to get ahead of accreditation in ISO15189 by applying to ENAO. To accredit or not to accredit, that is the question. The reason to start the process of accreditation would be interest in improvement of quality of lab services with better documentation of processes and responsibilities or interest of management. Through this journey we comprehend that the first step before accreditation is building enthusiastic team with education on quality management system. Other steps include selection of methods, developing or improving the metrology system, definition and structure of documents, preparation of a quality manual, SOPs, increasing staff competency etc. After this long journey our laboratory, AAHRL accredited in accordance with the requirement of ISO/IEC 15189 by ENAO with the scope of heamatology (HGB, WBC, RBC, PLT), Immunohematology (CD4 Absolute Count), and molecular biology(DNA-PCR for EID: Early HIV1 AIDS Infant Diagnosis). From this success we are beneficiary; for instances Staff technical competency highly praised, great boost to team, increase the team spirit, sense of great achievement and pride within accreditation [3]. Hence, higher quality laboratory testing associated with accreditation is expected to improve patient care by aiding the timeliness and accuracy of medical decision making [4]. Accreditation programs can help drive improvements in the management of individual laboratories and laboratory networks and may also have positive spillover effects on the performance in other sectors of the health care system [5]. It illustrates and can be summarized as leadership, vision and hard work brings to Laboratory Accreditation. Hence, ISO 15189 Laboratory Accreditation is more likely to be possible and can achieve in developing countries like Ethiopia.
